# The prognostic values of lymph node ratio for gynecological cancer: a systematic review and meta-analysis

**DOI:** 10.3389/fonc.2024.1475348

**Published:** 2024-10-01

**Authors:** Mengmeng Chen, Yisi Wang, Yali Chen, Ling Han, Ai Zheng

**Affiliations:** ^1^ Department of Obstetrics and Gynecology, West China Second University Hospital, Sichuan University, Chengdu, Sichuan, China; ^2^ Key Laboratory of Birth Defects and Related Diseases of Women and Children (Sichuan University), Ministry of Education, Sichuan, China

**Keywords:** lymph node ratio, gynecological cancer, prognosis, systematic review, meta-analysis

## Abstract

**Background:**

The aim of this study was to determine the relationship between the lymph node ratio (LNR) and the prognostic values of gynecological cancer.

**Materials and methods:**

PubMed, Web of Science, Embase, and the Central Cochrane Library were used to search for studies on LNR and gynecological cancer published before 18 April 2024. The effect measure for meta-analysis of primary outcomes was the hazard ratio (HR) for overall survival (OS), progression-free survival (PFS), and disease-free survival (DFS). Pooled HRs and 95% confidence intervals (CIs) were calculated using random- or fixed-effects models. Sensitivity analysis was applied to evaluate the robustness of the results. The *I* ^2^ statistic was used to measure heterogeneity. Subgroup analysis and meta-regression were chosen to illustrate the potential heterogeneity of the risk factors for outcomes. Publication bias was assessed using Egger’s test and Begg’s funnel plots.

**Results:**

A total of 34 studies with 23,202 cases were included in this meta-analysis. A meta-analysis found that higher LNR was associated with worse OS (HR = 2.42, 95% CI: 2.07–2.83; *I* ^2^ = 77.4%, *p* < 0.05), PFS (HR = 1.97, 95% CI: 1.66-2.32; *I* ^2^ = 0.00%, *p* > 0.05), and DFS (HR = 3.18, 95% CI: 2.12–4.76; *I* ^2^ = 64.3%, *p* < 0.05). Moreover, meta-analysis revealed significant differences in the association between LNR and OS of cervical cancer (CC) (HR = 2.53, 95% CI: 1.94–3.31; *I* ^2^ = 72.6%, *p* < 0.05), ovarian cancer (OC) (HR = 2.05, 95% CI: 1.66–2.54; *I* ^2^ = 76.7%, *p* < 0.05), endometrial cancer (EC) (HR = 2.16, 95% CI: 1.48–3.16; *I* ^2^ = 53.6%, *p* < 0.05), and vulvar cancer (VC) (HR = 8.13, 95% CI: 3.41–19.43; *I* ^2^ = 57.2%, *p* < 0.05).

**Conclusion:**

We observed a clear association between higher LNR and poorer prognosis in our study of patients with gynecological cancer. Further prospective studies are warranted to determine the optimal LNR and whether LNR can guide adjuvant therapy use in gynecological cancer. It is essential to conduct further prospective studies to establish the optimal LNR threshold, determine the minimum threshold of lymph node removal, and investigate whether LNR can serve as a reliable marker for guiding adjuvant therapy choices in gynecological cancer.

**Systematic review registration:**

https://www.crd.york.ac.uk/PROSPERO/#recordDetails, CRD42024541187.

## 1 Introduction

Lymph node metastasis is a common occurrence in gynecological cancers and has a significant impact on patient prognosis. However, the number of positive nodes during pelvic lymphadenectomy can be influenced by surgical technique and the accuracy of pathological examination. To overcome potential confounding effects, the use of lymph node ratio (LNR) has been proposed. LNR calculates the ratio of positive lymph nodes to the total number of resected lymph nodes and provides a more accurate representation of pelvic lymph node metastasis status. It has been identified as an independent predictor of survival in various cancers, including colon cancer (1), oral cancer (2), pancreatic cancer (3), breast cancer (4), esophageal cancer (5), and lung cancer (6).

Recently, there has been interest in using LNR as a prognostic tool in gynecologic malignancies such as cervical cancer (CC), ovarian cancer (OC), endometrial cancer (EC), and vulvar cancer (VC). However, the conclusions of studies in this area are not consistent. To address this, we conducted a systematic search of scientific databases to identify relevant publications and to explore the relationship between lymph node ratio and overall survival (OS), progression-free survival (PFS), and disease-free survival (DFS) in gynecological cancers.

## 2 Materials and methods

### 2.1 Protocol registration

This meta-analysis was performed in accordance with the Preferred Reporting Items for Systematic Reviews and Meta-Analyses (PRISMA) guidelines (7). Before data extraction, the review was registered with the International Prospective Register of Systematic Reviews (PROSPERO, Registration Number CRD42024541187).

### 2.2 Data collection

PubMed, Web of Science, Embase, and the Central Cochrane Library were used to search for studies on LNR and gynecological cancer published before 18 April 2024. The following keywords were used for literature retrieval: (“lymph node ratio” or “Ratio, Lymph Node”) and (“Uterine Cervical Neoplasms” or “Neoplasm, Uterine Cervical “ or “Ovarian Neoplasms” or “Neoplasm, Ovary” or “Neoplasm, Endometrial” or “Endometrial Neoplasms” or “Vulvar Neoplasms” or “Neoplasm, Vulva”). Additionally, the references in the obtained papers were examined to find any other relevant research outside of these two key phrases in the query.

### 2.3 Eligibility criteria and exclusion criteria

Inclusion criteria were as follows: (1) studies that investigated the relationship between LNR and OS, PFS, or DFS; (2) studies with CC, OC, EC, and VC confirmed by pathology; (3) studies that reported a hazard ratio (HR) with 95% CI for OS, PFS, or DFS; and (4) full articles published in English.

Exclusion criteria were as follows: (1) useful data could not be extracted; (2) the survival data or 95% confidence interval (CI) were not reported; and (3) editorials, reviews, and comments. In addition, when the data of a patient were used in multiple studies, we selected the most recent study.

### 2.4 Data extraction of data and quality assessment

The data extracted mainly included the following: the first author, publication date, sample size, cancer type, country, average age, duration of follow-up, cutoff value for LNR, and patient outcomes, including OS, PFS, and DFS.

In this meta-analysis, the quality assessment for the non-randomized studies was evaluated by two reviewers independently based on the Newcastle–Ottawa quality assessment scale (NOS) (8). The NOS was based on three categories: selected cases, comparability between groups, and outcome assessment. A score ≥ 6 was considered high-quality literature, to be included in our study.

### 2.5 Main outcomes

OS refers to the period from the date of the initial therapy to the date of all-cause mortality. PFS refers to the time from the date of the initial therapy to the date of disease progression, which was the time following successful treatment without disease progression or symptoms. DFS refers to the time from surgery to the last follow-up with no evidence of recurrence or distant metastasis.

### 2.6 Statistical analysis

We used STATA 15.0 software (StataCorp LP, College Station, TX, United States) to pool the extracted data for this meta-analysis. Hazard ratios with 95% CI were collected from individual studies, then combined using a random- or fixed-effects model, and finally presented in forest plots. Statistical heterogeneity was quantified by *I* ^2^ statistics. A random-effects model was used if there was prominent heterogeneity (*p* < 0.1 or *I* ^2^ > 50%); otherwise, a fixed-effects model was adopted (*p* > 0.1 or *I* ^2^ < 50%) (9). Sensitivity analysis was used to determine the robustness and stability of the results by calculating the heterogeneity in each situation in which a single study was removed in turn to evaluate the effect of a single study on the overall outcome. The risk of publication was assessed by visual inspection of Begg’s funnel plot and Egger’s linear regression test. In these two-tailed statistical tests, *p* < 0.05 (95% CI) was regarded as statistically significant. Meta-regression analysis, subgroup analysis, and publication bias were evaluated in analyses that included more than 10 studies.

## 3 Results

### 3.1 Study selection

**Figure 1:**
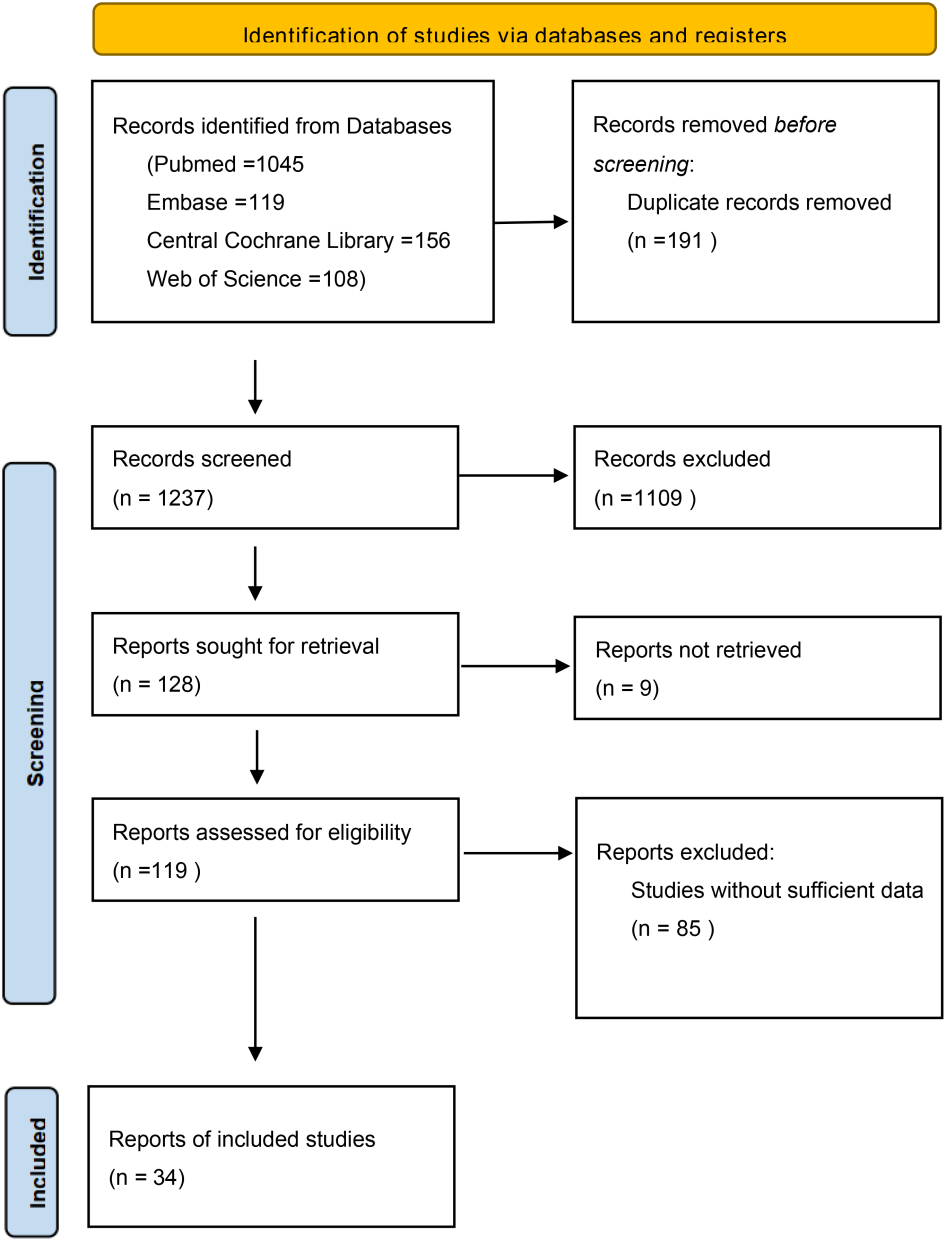
Flow diagram of the included studies.

As shown in **Figure 1**, through electronic searching on PubMed, Web of Science, Embase, and the Central Cochrane Library, 1,428 potential articles were screened. After excluding duplicate studies there were 1,237 records. Then, the titles and abstracts were screened, and 1,109 publications were removed as irrelevant. Finally, 119 full-text articles were identified for qualification, and 85 ineligible papers were eliminated because they did not provide primary outcome measurements. In the end, a total of 34 studies with 23,202 cases were eligible for the current meta-analysis.

### 3.2 Characteristics of the included studies

The main characteristics of the included studies are presented in **Table 1**. In this study, all necessary data were extracted from 34 studies from different countries including China (*n* = 10), Türkiye (*n* = 6), USA (*n* = 3), Germany (*n* = 3), Poland (*n* = 3), Republic of Korea (*n* = 2), Austria (*n* = 2), Thailand (*n* = 1), Brazil (*n* = 1), Tunisia (*n* = 1), Netherlands (*n* = 1), and France (*n* = 1). All included studies were retrospective studies. Among these articles, 14 studies involved 6,289 patients diagnosed with CC, 9 studies involved 15,058 patients diagnosed with OC, 8 studies involved 1,017 patients diagnosed with EC, and 3 studies involved 838 patients diagnosed with VC. All studies were retrospective cases, and all were rated seven or more stars according to the NOS criteria (**Table 2**).

**Table 1:** Main characteristics of the included literature.

First author	Year	Country	Recruitment period	Size	Cancer type	Age	Mean/median months of follow-up	Survival analysis	Cutoff LNR	NOS score
Ying Chen (10)	2013	China	NM	93	Cervical cancer	46	67	OS	0.05 0.2	8
Qinhao Guo (11)	2020	China	2006–2014	928	Cervical cancer	46.58	35.7	OS PFS	0.16	7
Juan Zhou (12)	2016	China	1988–2010	2,269	Cervical cancer	43	78	OS	0.16	8
Se lk Kim (13)	2021	Republic of Korea	2010–2018	55	Cervical cancer	52.6	NM	DFS	0.08831	7
S Polterauer (14)	2012	Austria	1996–2009	139	Cervical cancer	47.9	45.7	OS	Continuous	7
Xiang Fan (15)	2023	China	2012–2017	102	Cervical cancer	NM	63	OS DFS	0.3	8
Dan Li (16)	2019	China	2008–2013	1,435	Cervical cancer	47	77	OS	0.19	8
Chen Li (17)	2016	China	2007–2009	198	Cervical cancer	44	NM	OS DFS	0.2	7
Juan Zhou (18)	2015	China	1980–2012	60	Cervical cancer	37	30.5	OS	Continuous	7
Yoon Hee Lee (19)	2021	Republic of Korea	2007–2016	49	Cervical cancer	48.5	58	OS DFS	0.0625	7
Nicole D. Fleming (20)	2015	USA	1990–2011	95	Cervical cancer	39.7	64.8	OS PFS	0.076	8
S Polterauer (21)	2010	Austria	1995–2008	88	Cervical cancer	49.9	37.1	OS DFS	0.1	7
Koray Aslan (22)	2020	Türkiye	2006–2018	185	Cervical cancer	50	45.5	OS DFS	0.05	7
E Olthof (23)	2021	Netherlands	1995–2020	593	Cervical cancer	NM	NM	OS	0.177	7
Koray Aslan (24)	2020	Türkiye	1997–2017	62	Ovarian cancer	47	45	OS PFS	0.09	7
Ali Ayhan (25)	2018	Türkiye	2007–2016	229	Ovarian cancer	56	36	OS	0.1 0.5	7
Xiaoxia Tong (26)	2019	China	1973–2013	7,819	Ovarian cancer	NM	NM	OS	0.42	7
Dan Nie (27)	2019	China	2008–2014	265	Ovarian cancer	56	40	OS DFS	0.25	7
Beyhan Ataseven (28)	2014	Germany	2000–2013	398	Ovarian cancer	NM	45	OS	0.25	7
Katarzyna Lepinay (29)	2020	Poland	2010–2015	144	Ovarian cancer	NM	NM	OS	0.1	7
Juan Zhou (30)	2016	China	1990–2012	5,926	Ovarian cancer	59	33	OS	0.42	7
M.A. Ayadi (31)	2018	Tunisia	2000–2010	84	Ovarian cancer	54	65	OS	0.5	8
P. Widschwendter (32)	2017	Germany	2000–2012	131	Ovarian cancer	NM	NM	OS	Continuous	7
Katarzyna Gorzelnik (33)	2022	Poland	2000–2015	75	Endometrial cancer	60	NM	OS	0.3	7
Nicole D. Fleming (34)	2015	Brazil	2000–2011	124	Endometrial cancer	60	49.4	OS PFS	0.4 (OS) 0.5 (PFS)	7
Ali Ayhan (25)	2017	Türkiye	1998–2016	207	Endometrial cancer	58	40	OS PFS	0.15	7
Siriwan Tangjitgamol (35)	2019	Thailand	1995–2017	82	Endometrial cancer	59.5	NM	PFS	0.1	7
Stephan Polterauer (36)	2012	Now York	1993–2008	216	Endometrial cancer	65.5	30.5	OS PFS	0.1 0.5	7
Nicole D. Fleming (20)	2015	USA	1990–2011	95	Endometrial cancer	39.7	64.8	OS PFS	0.66 (OS) 0.076 (PFS)	8
Tayfun Toptas (37)	2015	Türkiye	2005–2013	38	Endometrial cancer	32.5	64	OS PFS	0.065	8
B. Akkus Yildirim (38)	2018	Türkiye	2001–2016	180	Endometrial cancer	60	50.5	OS PFS	0.1	7
Stephan Polterauer (39)	2017	Germany	NM	370	Vulvar cancer	64.5	26.4	OS DFS	0.1 0.2	7
E. Serre (40)	2019	France	2005–2015	176	Vulvar cancer	68.7	NM	OS	0.2	7
Stephan Polterauer (41)	2020	Poland	2001–2005	292	Vulvar cancer	69.9	NM	OS	0.2	7

**Table 2:** Quality of the included studies.

Study		Selection			Comparability			Outcome		Total
	Representativeness	Selection of non-exposed	Ascertainment of exposure	Outcome not present at the start	Comparability of most important factors	Comparability on other risk factors	Assessment of outcome	Long enough follow-up (median ≥ 5 years)	Adequacy (completeness of follow-up)	
Ying Chen (10)	√	√	√	√	√	×	√	√	√	8
Qinhao Guo (11)	√	√	√	√	√	×	√	×	√	7
Juan Zhou (12)	√	√	√	√	√	×	√	√	√	8
Se lk Kim (13)	√	√	√	√	√	×	√	×	√	7
S Polterauer (14)	√	√	√	√	√	×	√	×	√	7
Xiang Fan (15)	√	√	√	√	√	×	√	√	√	8
Dan Li (16)	√	√	√	√	√	×	√	√	√	8
Chen Li (17)	√	√	√	√	√	×	√	×	√	7
Juan Zhou (18)	√	√	√	√	√	×	√	×	√	7
Yoon Hee Lee (19)	√	√	√	√	√	×	√	×	√	7
Nicole D. Fleming (20)	√	√	√	√	√	×	√	√	√	8
S Polterauer (21)	√	√	√	√	√	×	√	×	√	7
Koray Aslan (22)	√	√	√	√	√	×	√	×	√	7
E. Olthof (23)	√	√	√	√	√	×	√	×	√	7
Koray Aslan (24)	√	√	√	√	√	×	√	×	√	7
Ali Ayhan (25)	√	√	√	√	√	×	√	×	√	7
Xiaoxia Tong (26)	√	√	√	√	√	×	√	×	√	7
Dan Nie (27)	√	√	√	√	√	×	√	×	√	7
Beyhan Ataseven (28)	√	√	√	√	√	×	√	×	√	7
Katarzyna Lepinay (29)	√	√	√	√	√	×	√	×	√	7
Juan Zhou (30)	√	√	√	√	√	×	√	×	√	7
M.A. Ayadi (31)	√	√	√	√	√	×	√	√	√	8
P. Widschwendter (32)	√	√	√	√	√	×	√	×	√	7
Katarzyna Gorzelnik (33)	√	√	√	√	×	×	√	×	√	7
Nicole D. Fleming (34)	√	√	√	√	√	×	×	×	×	7
Ali Ayhan (25)	√	√	√	√	√	×	×	×	×	7
Siriwan Tangjitgamol (35)	√	√	√	√	√	×	×	×	×	7
Stephan Polterauer (36)	√	√	√	√	√	×	×	×	×	7
Nicole D Fleming (20)	√	√	√	√	√	×	×	√	√	8
Tayfun Toptas (37)	√	√	√	√	√	×	√	√	√	8
B. Akkus Yildirim (38)	√	√	√	√	√	×	√	×	√	7
Stephan Polterauer (39)	√	√	√	√	√	×	√	×	√	7
E. Serre (40)	√	√	√	√	√	×	√	×	√	7
Stephan Polterauer (41)	√	√	√	√	√	×	√	×	√	7

"√" indicates that the criteria are met, while "×" indicates that the criteria are not met.

### 3.3 Meta-analysis

#### 3.3.1 Primary outcomes

##### 3.3.1.1 LNR and OS

**Figure 2:**
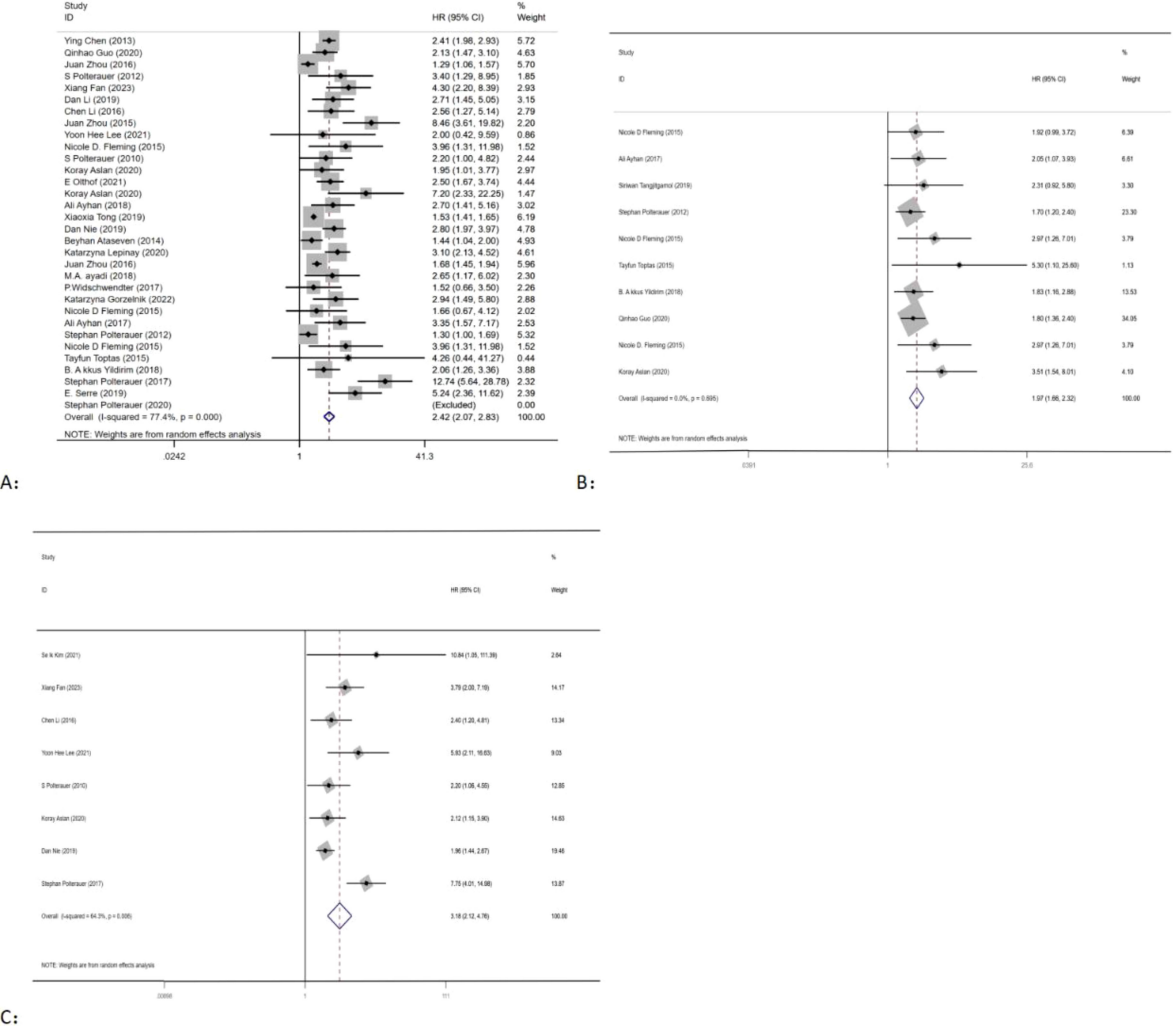
Forest plots and pooled estimates of the effect for meta-analysis of the association between LNR and OS **(A)**, PFS **(B)**, and DFS **(C)** in patients with gynecological cancer.

Out of the 34 (10–41) eligible studies, 32 (10–12, 14–34, 36–41) studies, namely, 13 (10–12, 14–23) studies with CC, 9 (24–32) studies with OC, 7 (20, 25, 33, 34, 36–38) studies with EC, and 3 (39–41) studies with VC, analyzed the association between LNR and OS. Using a random-effects model, the pooled results of HR and OS statistics from these 32 studies showed that higher levels of LNR were associated with worse OS (HR = 2.42, 95% CI: 2.07–2.83; *I* ^2^ = 77.4%, *p* < 0.05), as shown in (**Figure 2A**). However, the results also indicated a high heterogeneity between studies (*I* ^2^ = 77.4%, *p* < 0.05).

##### 3.3.1.2 LNR and PFS

Seven studies (20, 25, 34–38) with EC, two studies (11, 15) with CC, and one study (24) with OC explored the association between LNR and PFS. Using a fixed-effects model, pooled results of HR and PFS statistics from 10 studies indicated that higher LNR levels were associated with worse PFS (HR = 1.97, 95% CI: 1.66–2.32; *I* ^2^ < 50%, *p* > 0.05) (**Figure 2B**). The results showed that there was low heterogeneity between studies (*I* ^2^ < 50%, *p* > 0.05).

##### 3.3.1.3 LNR and DFS

Eight studies, consisting of six studies (13, 15, 17, 19, 21, 22) with CC, one study (24) with OC, and one study (39) with VC, were included in the analysis of the association between LNR and PFS. Using a random-effects model, the pooled results indicated that higher levels of LNR were associated with worse DFS (HR = 3.18, 95% CI: 2.12–4.76; *I* ^2^ = 64.3%, *p* < 0.05) (**Figure 2C**). However, there was high heterogeneity between studies (*I* ^2^ > 50%, *p* < 0.05).

#### 3.3.2 Subgroup and meta-regression analysis

**Figure 3:**
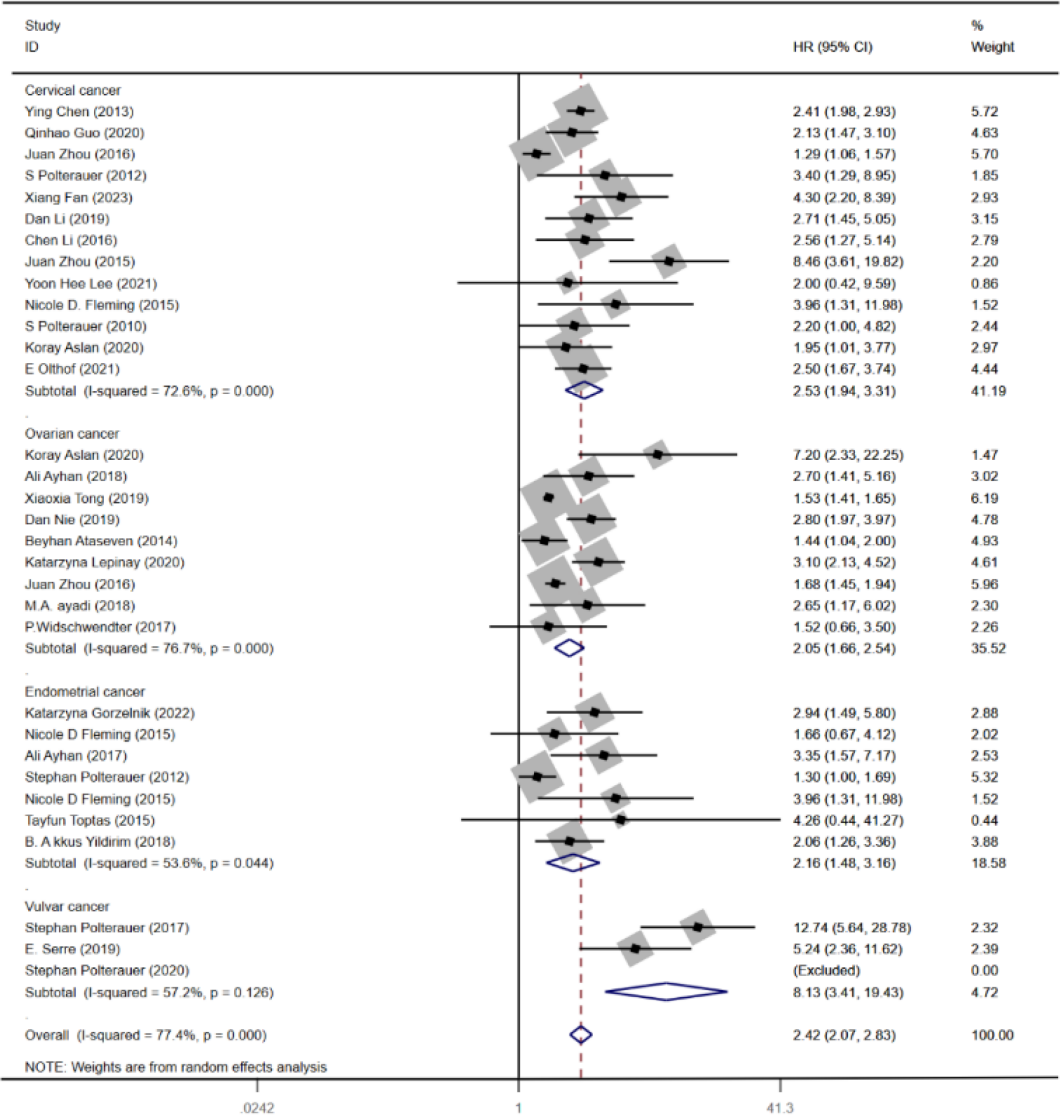
Forest plot for subgroup analysis for OS in gynecological cancer.

As the outcome of OS shows high heterogeneity, both meta-regression and subgroup analyses were conducted to explore the factors contributing to the high heterogeneity. Subgroup analysis was carried out based on the type of cancer. The analysis revealed significant differences in the association between LNR and OS of CC (HR = 2.53, 95% CI: 1.94–3.31; *I* ^2^ = 72.6%, *p* < 0.05), OC (HR = 2.05, 95% CI: 1.66–2.54; *I* ^2^ = 76.7%, *p* < 0.05), EC (HR = 2.16, 95% CI: 1.48–3.16; *I* ^2^ = 53.6%, *p* < 0.05), and VC (HR = 8.13, 95% CI: 3.41–19.43; *I* ^2^ = 57.2%, *p* < 0.05) (**Figure 3**). Furthermore, meta-regression analysis was carried out to investigate possible sources of heterogeneity. Single covariate regression was performed using variables such as country, sample size, type of gynecological cancer, and publication year. The results indicated that sample size was the main factor contributing to the heterogeneity, with a *p*-value of 0.014 (**Table 3**).

**Table 3:** Results of the meta-regression.

Co-factor	Coefficient	95% confidence interval	*p*-value
Year	0.055	(−0.003,0.114)	0.062
Type	0.193	(−0.063,0.449)	0.133
Country	−0.045	(−0.130,0.038)	0.274
Size	−0.00	(−0.00,−0.000)	0.014

We did not conduct meta-analyses, and subgroup analyses for PFS and DFS, as they c.

#### 3.3.3 Sensitivity analysis

**Figure 4:**
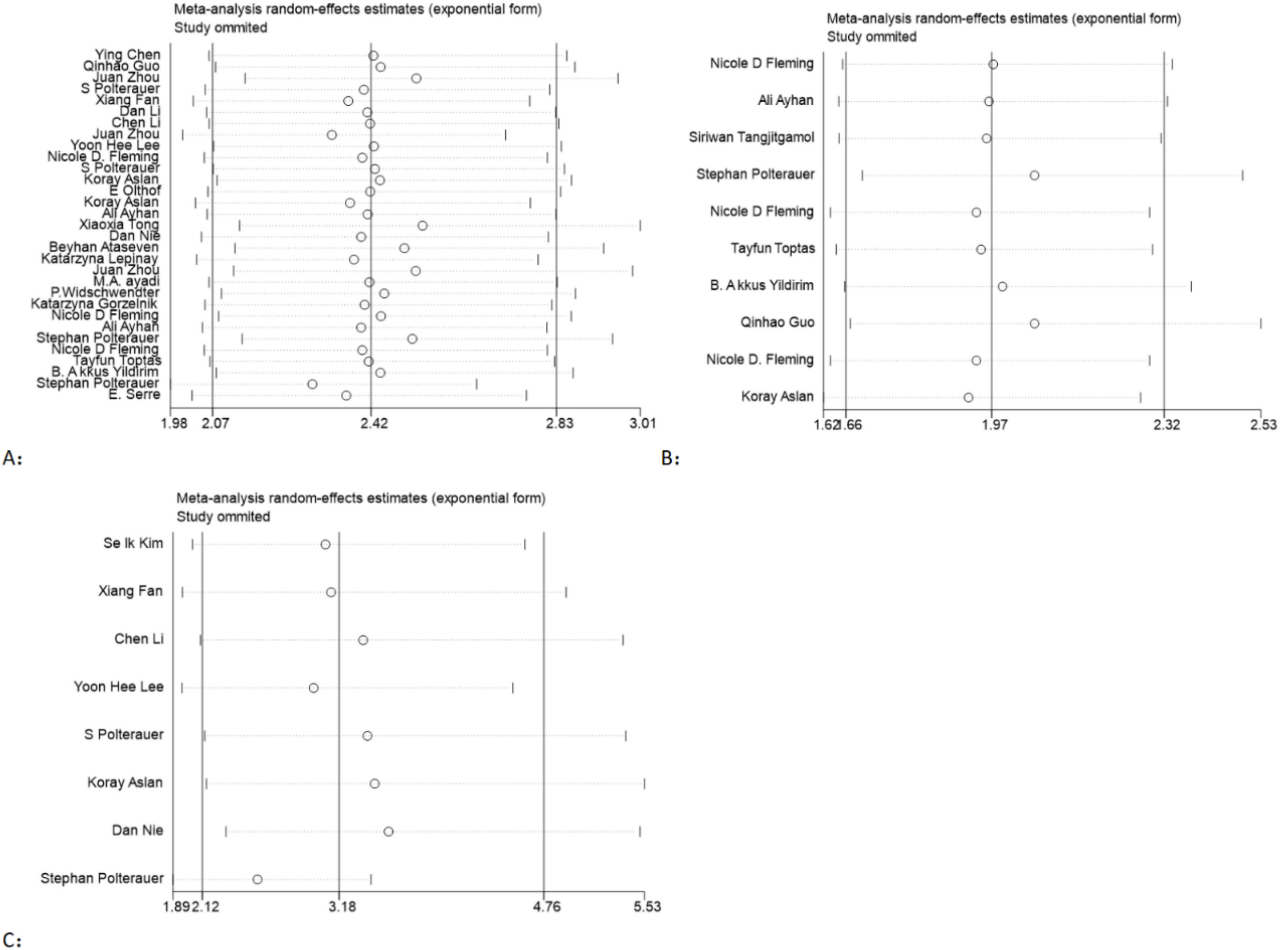
Sensitivity analysis of the association between LNR and OS **(A)**, PFS **(B)**, and DFS **(C)** in gynecological cancer.

In order to assess the stability of the models, a sensitivity analysis was conducted by excluding each individual study and calculating new HRs. The results showed that the HRs were relatively stable, as illustrated in **Figure 4**.

#### 3.3.4 Publication bias

**Figure 5:**
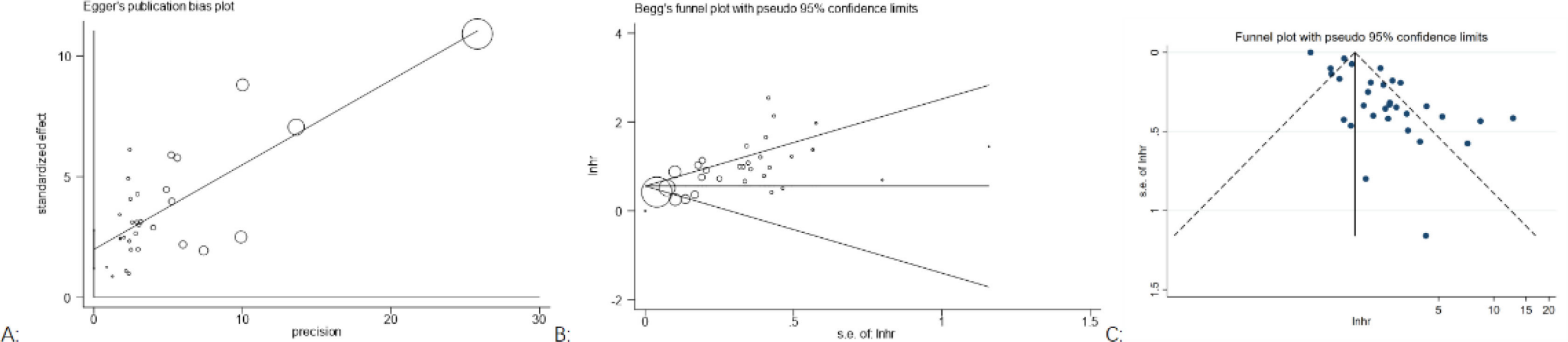
Egger’s **(A)**, Begg’s **(B)**, and funnel plots **(C)** show bias for OS.

Publication bias was assessed by Begg’s funnel plots and Egger’s test. For OS, the *p*-values for Egger’s test (**Figure 5A**) and Begg’s test (**Figure 5B**) were *p* = 0.255 and *p* < 0.00, respectively. Visual inspection of Begger’s funnel plot (**Figure 5C**) was not symmetrical, suggesting evidence of publication bias. We did not conduct a publication bias analysis for PFS and DFS, as they did not include more than 10 studies.

## 4 Discussion

This meta-analysis revealed that higher LNR was significantly associated with poorer prognosis across multiple metrics. Specifically, it found that higher LNR was correlated with worse OS, PFS, and DFS, indicating that patients with higher LNR often have worse outcomes. Furthermore, the pooled HRs for OS across different types of cancers are 3.42 for CC, 2.05 for OC, 2.16 for EC, and 8.13 for VC, suggesting that higher LNR often correlates with worse survival outcomes in CC, OC, EC, and VC. These findings underscore the significant prognostic implications of LNR in gynecological cancer, highlighting its role as a critical factor in predicting patient outcomes.

For CC, based on the results of previous studies, the cutoff value of LNR in patients with CC ranges from 5% to 30% (9–22), with higher LNR consistently associated with poorer prognosis. A meta-analysis (42) conducted in 2017, comprising eight articles with 3,325 patients diagnosed with CC, confirmed that higher LNR was an unfavorable prognostic factor for OS and DFS, which is consistent with our findings. In 2018, the FIGO Committee added IIICI (pelvic lymph node metastasis only) and IIIC2 (para-aortic lymph node metastasis) to the FIGO staging system for CC (43). Nevertheless, this was solely based on anatomic location and did not consider other lymph node characteristics. Researchers have looked for a reliable lymph node measure in CC, yielding conflicting results. Yoon et al. (19) showed that LNR was the most robust biomarker for predicting tumor recurrence, while Qinhao et al. (11) found that positive lymph nodes had the best prognostic performance for OS and PFS. Therefore, further studies are warranted to explore this aspect and establish the most reliable measure.

In OC, previous studies have identified varying cutoff values for LNR ranging from 9% to 50% (23–31). Consistently, higher LNR was linked to poorer prognosis, which is in line with our study findings. It is worth noting that OC is a heterogeneous group of diseases with varying histology, molecular genetic analysis, and prognosis (44); some researchers have begun to conduct more in-depth research on the classification of epithelial OC. The prognostic value of lymph node ratio has been confirmed in high-grade serous ovarian carcinoma (45), low-grade serous ovarian carcinoma (22), clear cell ovarian carcinoma (27), and borderline ovarian tumors (46). Especially in borderline ovarian tumors (46), David controlled for age, histology, stage, tumor size, and adequate lymphadenectomy status, and LNR remained an independent factor for survival; qualitative assessment of lymph node involvement is not a prognostic factor for survival. Another study (30) based on the Surveillance, Epidemiology, and End Results database showed that there is a significant and independent correlation between higher LNR and poorer OS, and its prognostic value is superior to removed lymph nodes and positive lymph node counts. Despite this, a study conducted by Xiao et al. (26) found that LNR did not reach statistical significance for discriminating OS in stage IV patients, although it showed better performance than the number of positive lymph nodes. Therefore, future research should emphasize the prognostic utility of LNR in various stages of OC.

In EC, according to prior research (20, 25, 32–37), the cutoff values for LNR in EC patients span from 6.5% to 50%. These studies consistently indicated that higher LNR is associated with a worse prognosis, a trend observed in our study. Previous multi-center retrospective studies (20, 25, 32–37) have found a correlation between LNR and worse OS and PFS. Xi-Lin et al. (47) found that the lymph node ratio had a better predictive performance for these patients than the number of removed lymph nodes, the number of positive lymph nodes, and the number of negative lymph nodes. However, a study conducted by Fleming et al. (34) did not find a statistically significant association between LNR and OS, probably due to the small patient cohort in this single-institution study and the low median count of retrieved lymph nodes. This suggests that the prognostic value of LNR may be limited to patients who have undergone a minimum threshold of lymph node removal. Nevertheless, this threshold has not been universally adopted as a clinical standard. Future research should focus on identifying optimal lymph node removal strategies to mitigate the morbidity associated with systemic lymphadenectomy.

In VC, Kunos et al. (48) initially described LNR for prognostic assessment in patients with VC, and they found that patients with LNR > 20% had an increased likelihood of contralateral positive lymph nodes, recurrence, and cancer-specific death compared with patients with LNR < 20%. Moreover, some studies (38–40) stratified patients into three groups (LNR = 0%, 0% < LNR < 20%, and LNR > 20%), and the LNR > 20% group had the highest risk for OS and recurrence, which is consistent with our study. According to a study conducted by Polterauer et al., LNR appears to be a consistent, independent prognostic parameter for both OS and PFS, and its predictive value is superior to positive lymph node number. These studies support the predictive value of the LNR for VC.

Our research has certain strengths. First, this is the first complete meta-analysis to quantify the role of LNR in the prognosis of gynecological cancer. Second, this meta-analysis included a large number of primary studies (34 papers) and patients (23,202 patients with positive lymph nodes), which allowed for a more robust statistical analysis. Finally, our findings have demonstrated the importance of LNR in the prognosis of gynecological cancer. Therefore, we recommend LNR as a prognostic parameter that should be included in a future gynecological cancer staging system.

While our study has shown that LNR is of significant prognostic value in gynecologic cancers, it also has limitations. First, literature-based meta-analyses rely on published data and may be biased toward positive results, and we found that it does have bias in our study. In addition, the absence of data on tumor size, pathological stages, number of examined lymph nodes, number of metastasized lymph nodes, and surgical methods limited further subgroup analysis. Furthermore, the LNR cutoff in different studies was inconsistent. Finally, all included studies were retrospective, and this study type has intrinsic limitations. Thus, more prospective data are required to further ascertain the prognostic value of LNR in specific populations.

## 5 Conclusion

Higher LNR is linked to lower OS, PFS, and DFS in patients diagnosed with gynecological cancer. The prognostic value of LNR for OS is consistent across different types of gynecological cancer, including CC, OC, EC, and VC. Further prospective studies are essential to establish the optimal LNR threshold, determine the minimum threshold of lymph node removal, and evaluate whether LNR can effectively guide the use of adjuvant therapies in gynecological cancer.

## Data Availability

The original contributions presented in the study are included in the article/supplementary material. Further inquiries can be directed to the corresponding authors.

## References

[1] Ichhpuniani S McKechnie T Lee JY Biro J Lee Y Park L . Lymph node ratio as a predictor of survival for colon cancer: A systematic review and meta-analysis. Am Surgeon. (2024) 90:840–50. doi: 10.1177/00031348231209532 37967460

[2] Gartagani Z Doumas S Kyriakopoulou A Economopoulou P Psaltopoulou T Kotsantis I . Lymph node ratio as a prognostic factor in neck dissection in oral cancer patients: A systematic review and meta-analysis. Cancers. (2022) 14(18). doi: 10.3390/cancers14184456 PMC9497248 36139617

[3] Karjol U Chandranath A Jonnada P Cherukuru S Annavarjula V Morla SA . Lymph node ratio as a prognostic marker in pancreatic cancer survival: A systematic review and meta-analysis. Cureus J Med Sci. (2020) 12(8). doi: 10.7759/cureus.9597 PMC7417066 32789099

[4] Liu JZ Li YF Zhang WF Yang CH Yang C Chen L . The prognostic role of lymph node ratio in breast cancer patients received neoadjuvant chemotherapy: A dose-response meta-analysis. Front Surg. (2022) 9. doi: 10.3389/fsurg.2022.971030 PMC9644128 36386510

[5] Song JN Zhang H Jian JL Chen H Zhu XD Xie JF . The prognostic significance of lymph node ratio for esophageal cancer: A meta-analysis. J Surg Res. (2023) 292:53–64. doi: 10.1016/j.jss.2023.07.027 37586187

[6] Zhou J Lin ZY Lyu MY Chen N Liao H Wang ZH . Prognostic value of lymph node ratio in non-small-cell lung cancer: a meta-analysis. Japanese J Clin Oncol. (2020) 50:44–57. doi: 10.1093/jjco/hyz120 31735973

[7] Moher D Shamseer L Clarke M Ghersi D Liberati A Petticrew M . PRISMA-P Group.Preferred reporting items for systematic review and meta-analysis protocols (PRISMA-P) 2015 statement. Syst Rev. (2015) 4:1. doi: 10.1186/2046-4053-4-1 25554246 PMC4320440

[8] Stang A . Critical evaluation of the Newcastle-Ottawa scale for the assessment of the quality of nonrandomized studies in meta-analyses. Eur J Epidemiol. (2010). 65:603–5. doi: 10.1186/2046-4053-4-1 20652370

[9] DerSimonian R Laird N . Meta-analysis in clinical trials revisited. Contemp Clin Trials. (2015) 45:139–45. doi: 10.1016/j.cct.2015.09.002 PMC4639420 26343745

[10] Chen Y Zhang L Tian J Ren XB Hao Q . Combining the negative lymph nodes count with the ratio of positive and removed lymph nodes can better predict the postoperative survival in cervical cancer patients. Cancer Cell Int. (2013) 13:6. doi: 10.1186/1475-2867-13-6 23374254 PMC3576300

[11] Guo Q Zhu J Wu Y Wen H Xia LF Yu M . Comparison of different lymph node staging systems in patients with node-positive cervical squamous cell carcinoma following radical surgery. J Cancer. (2020) 11:7339–47. doi: 10.7150/jca.48085 PMC7646177 33193898

[12] Zhou J Chen QH Wu SG He ZY Sun JY Li FY . Lymph node ratio may predict the benefit of postoperative radiotherapy in node-positive cervical cancer. Oncotarget. (2016) 7:29420–28. doi: 10.18632/oncotarget.v7i20 PMC5045406 27105541

[13] Kim SI Kim TH Lee M Kim HS Chung HH Lee TS . Lymph node ratio is a strong prognostic factor in patients with early-stage cervical cancer undergoing minimally invasive radical hysterectomy. Yonsei Med J. (2021) 62:231–39. doi: 10.3349/ymj.2021.62.3.231 PMC7934101 33635013

[14] Polterauer S Grimm C Hofstetter G Concin N Natter C Sturdza A . Nomogram prediction for overall survival of patients diagnosed with cervical cancer. Br J Cancer. (2012) 107:918–24. doi: 10.1038/bjc.2012.340 PMC3464766 22871885

[15] Fan X Wang Y Yang N Yang N Zhu PF . Prognostic analysis of patients with stage IIIC1p cervical cancer treated by surgery. World J Surg Oncol. (2023) 21:186. doi: 10.1186/s12957-023-03076-9 37344912 PMC10283242

[16] Li D Xu X Yan D Yuan SH Ni J Lou HM . Prognostic factors affecting survival and recurrence in patients with early cervical squamous cell cancer following radical hysterectomy. J Int Med Res. (2019) 48(4). doi: 10.1177/0300060519889741 PMC7607059 31889461

[17] Li C Liu W Cheng Y . Prognostic significance of metastatic lymph node ratio in Squamous cell carcinoma of the cervix. OncoTargets Ther. (2016) 9:3791–97. doi: 10.2147/OTT.S97702 PMC4922781 27382315

[18] Zhou J Sun JY Chen SY Li FY Lin HX Wu SC . Prognostic value of lymph node ratio in patients with small-cell carcinoma of the cervix based on data from a large national registry. OncoTargets Ther. (2015) 9:67–73. doi: 10.2147/OTT.S96206 PMC4694687 26730205

[19] Lee YH Chong GO Kim SJ Hwang JH Kim JM Park NJY . Prognostic value of lymph node characteristics in patients with cervical cancer treated with radical hysterectomy. Cancer Manag Res. (2021) 13:8137–45. doi: 10.2147/CMAR.S332612 PMC8560076 34737642

[20] Fleming ND Frumovitz M Schmeler KM Dos Reis R Munsell MF Eifel PJ . Significance of lymph node ratio in defining risk category in node-positive early stage cervical cancer. Gynecol Oncol. (2015) 136:48–53. doi: 10.1016/j.ygyno.2014.11.010 25451695 PMC4430191

[21] Polterauer S Hefler L Seebacher V Rahhal J Tempfer C Horvat R . The impact of lymph node density on survival of cervical cancer patients. Br J Cancer. (2010) 103:613–6. doi: 10.1038/sj.bjc.6605801 PMC2938249 20628380

[22] Aslan K Meydanli MM Oz M Tohma YA Haberal A Ayhan A . The prognostic value of lymph node ratio in stage IIIC cervical cancer patients triaged to primary treatment by radical hysterectomy with systematic pelvic and para-aortic lymphadenectomy. J Gynecol Oncol. (2020) 31:e1. doi: 10.3802/jgo.2020.31.e1 31788991 PMC6918892

[23] Olthof E Mom C Wenzel H Van Der Velden J Van Der Aa M . The prognostic value of the number of positive lymph nodes and the lymph node ratio in early stage cervical cancer. Int J Gynecol Cancer. (2021) 31:A45. doi: 10.1136/ijgc-2021-IGCS.108 PMC9564443 35218205

[24] Aslan K Meydanli MM Akilli H Durmus Y Gokcu M Kayikcioglu F . Does lymph node ratio have any prognostic significance in maximally cytoreduced node-positive low-grade serous ovarian carcinoma? Arch Gynecol Obstet. (2020) 302:183–90. doi: 10.1007/s00404-020-05580-9 32409929

[25] Ayhan A Ozkan NT Oz M Comert GK Cuylan ZF Coban G . Impact of lymph node ratio on survival in stage IIIC endometrioid endometrial cancer: a Turkish Gynecologic Oncology Group study. J Gynecol Oncol. (2018) 29(4). doi: 10.3802/jgo.2018.29.e48 PMC5981100 29770619

[26] Tong X Li H Chen H Zhai D Pang YY Lin RY . Prognostic significance of lymph node ratio in ovarian cancer. Open Med. (2019) 14:279–86. doi: 10.1515/med-2019-0024 PMC6419391 30886899

[27] Nie D Mao X Li Z . Prognostic value of lymph nodes ratio in patients with stage III ovarian clear cell carcinoma: A retrospective study of patients in Southwest China. J Cancer. (2019) 10:4689–94. doi: 10.7150/jca.29896 PMC6746121 31528234

[28] Ataseven B Grimm C Harter P Prader S Traut A Heitz F . Prognostic value of lymph node ratio in patients with advanced epithelial ovarian cancer. Gynecol Oncol. (2014) 135:435–40. doi: 10.1016/j.ygyno.2014.10.003 25312398

[29] Lepinay K Szubert S Lewandowska A . The association between lymph node metastases and long-term survival in patients with epithelial ovarian cancer. Contemp Oncol (Pozn). (2020) 24:163–71. doi: 10.5114/wo.2020.99029 PMC7670182 33235542

[30] Zhou J He Z-Y Li F-Y Sun JY Lin HX Wu SG . Prognostic value of lymph node ratio in stage IIIC epithelial ovarian cancer with node-positive in a SEER population-based study. Oncotarget. (2016) 7:7952–59. doi: 10.18632/oncotarget.6911 PMC4884966 26788911

[31] Ayadi MA Masouri H Ben safta I Sakhri S Hechiche M Ben Hassouna J . Impact of lymph node ratio on survival for epithelial ovarian cancer: A Tunisian study. Int J Gynecol Cancer. (2018) 28:648.

[32] Widschwendter P Schuh F DeGregorio N Friedl T Scholz C Bauer E . Lymph node ratio and outcome in ovarian cancer-a retrospective single center analysis. Int J Gynecol Cancer. (2017) 27:1007.

[33] Gorzelnik K Szubert S Knafel A Wojcikiewicz A Nowakowski B Koper K . An analysis of the significance of the lymph node ratio and extracapsular involvement in the prognosis of endometrial cancer patients. Wspolczesna Onkol. (2022) 26:144–49. doi: 10.5114/wo.2022.118243 PMC9319184 35903209

[34] Fleming ND Soliman PT Westin SN Dos Reis R Munsell M Klopp AH . Impact of lymph node ratio and adjuvant therapy in node-positive endometrioid endometrial cancer. Int J Gynecol Cancer. (2015) 25:1437–44. doi: 10.1097/IGC.0000000000000510 PMC4581897 26332387

[35] Tangjitgamol S Kittisiam T Sriraumpuch J . Impact of metastatic lymph node to total lymph node ratio on survival of endometrial cancer patients. Gynecol Obstet Invest. (2019) 84:463–71. doi: 10.1159/000497484 30836353

[36] Polterauer S Khalil S Zivanovic O Abu Rustum NR Hofstetter G Concin N . Prognostic value of lymph node ratio and clinicopathologic parameters in patients diagnosed with stage iiic endometrial cancer. Obstet Gynecol. (2012) 119:1210–18. doi: 10.1097/AOG.0b013e318255060c 22617586

[37] Toptas T Simsek T . Stage IIIC endometrial cancer: The need for novel subgrouping according to the ratio of metastatic lymph nodes. Arch Gynecol Obstet. (2015) 291:391–98. doi: 10.1007/s00404-014-3409-z 25115282

[38] Yildirim BA Onal C Sari SY Yavas G Gultekin M Guler OC . The utility of dissected lymph node number and lymph node metastasis ratio in stage IIIC endometrium adenocarcinoma: A multicentric analysis. Int J Radiat Oncol Biol Phys. (2018) 102:E642–E43. doi: 10.1016/j.ijrobp.2018.07.1750

[39] Polterauer S Schwameis R Grimm C Macuks R Iacoponi S Zalewski K . Prognostic value of lymph node ratio and number of positive inguinal nodes in patients with vulvar cancer. Gynecol Oncol. (2017) 147:92–7. doi: 10.1016/j.ygyno.2017.07.142 28797698

[40] Serre E Diguisto C Body G Raimond E Bendifallah S Touboul C . Prognostic significance of groin lymph node ratio in vulvar squamous cell carcinoma. Gynecol Obstet Fertil Senol. (2020) 48:729–35. 10.1016/j.gofs.2020.04.011 32339764

[41] Polterauer S Schwameis R Grimm C Hillemannsc P Jückstockd J Hilpert F . Lymph node ratio in inguinal lymphadenectomy for squamous cell vulvar cancer: Results from the AGO-CaRE-1 study. Gynecol Oncol. (2019) 153:286–91. doi: 10.1016/j.ygyno.2019.02.007 30760408

[42] Cui H Huang Y Wen W Li XD Xu DY Liu L . Prognostic value of lymph node ratio in cervical cancer: A meta-analysis. Med (United States). (2022) 101:E30745. doi: 10.1097/MD.0000000000030745 PMC9592518 36281189

[43] Wright JD Matsuo K Huang Y Tergas AI Hou JY Khoury-Collado F . Prognostic performance of the 2018 international federation of gynecology and obstetrics cervical cancer staging guidelines. Obstet Gynecol. (2019) 134:49–57. doi: 10.1097/AOG.0000000000003311 31188324 PMC7641496

[44] Prat J D’Angelo E Espinosa I . Ovarian carcinomas: at least five different diseases with distinct histological features and molecular genetics. Hum Pathol. (2018) 80:11–27. doi: 10.1016/j.humpath.2018.06.018 29944973

[45] Ayhan A Ozkan NT Sari ME Celik H Dede M Akbayir O . Impact of lymph node ratio on survival in stage III ovarian high-grade serous cancer: a Turkish Gynecologic Oncology Group study. J Gynecol Oncol. (2018) 29(1). doi: 10.3802/jgo.2018.29.e12 PMC5709522 29185270

[46] Nusbaum DJ Mandelbaum RS Machida H Matsuzaki S Roman LD Sood AK . Significance of lymph node ratio on survival of women with borderline ovarian tumors. Arch Gynecol Obstet. (2020) 301:1289–98. doi: 10.1007/s00404-020-05535-0 PMC7523228 32303888

[47] Yang X-L Huang N Wang MM Lai H Wu D-J . Comparison of different lymph node staging schemes for predicting survival outcomes in node-positive endometrioid endometrial cancer patients. Front Med (Lausanne). (2021) 8:688535. doi: 10.3389/fmed.2021.688535 34307415 PMC8298894

[48] Kunos C Simpkins F Gibbons H Chunqiao T Howard H . Radiation therapy compared with pelvic node resection for node-positive vulvar cancer: a randomized controlled trial. Obstet Gynecol. (2009) 114:537–46. doi: 10.1097/AOG.0b013e3181b12f99 19701032

